# Supplementary material for: Bio-Mechanism of Catechin as Pheromone Signal Inhibitor: Prediction of Antibacterial Agent Action Mode by In Vitro and In Silico Study
Source: Molecules. 2021 Oct 22;26(21):6381. doi: 10.3390/molecules26216381 (PMC8587927; doi:10.3390/molecules26216381)
Supplement: Supplementary file 1 [file molecules-26-06381-s001.zip › molecules-1405405-supplementary.pdf]

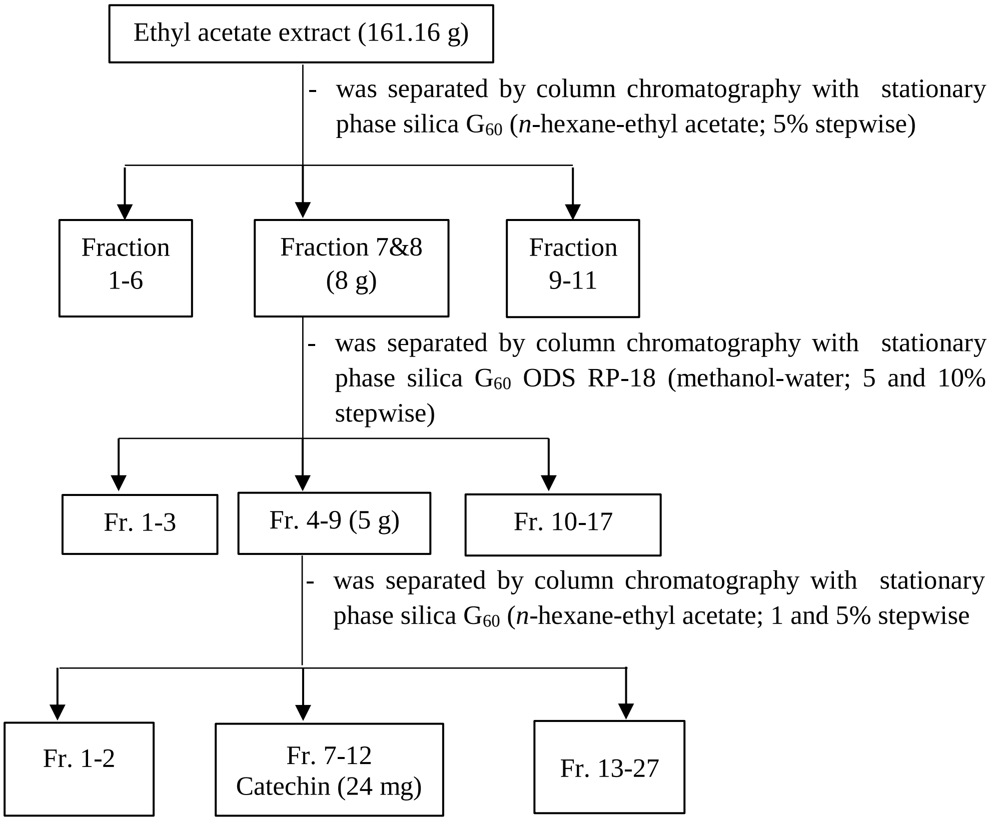

Figure S1. Scheme of isolation

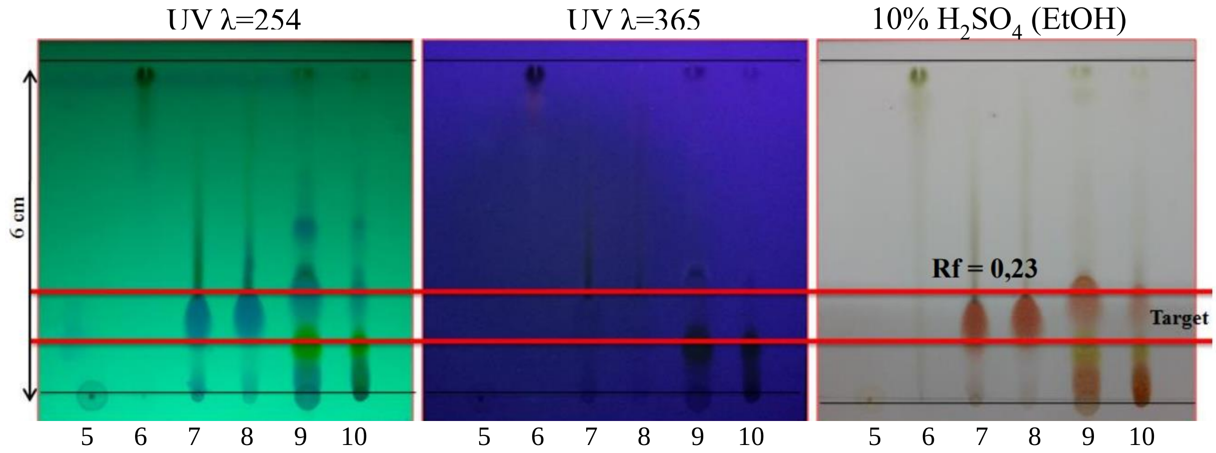

Figure S2. Chromatogram of the first separation

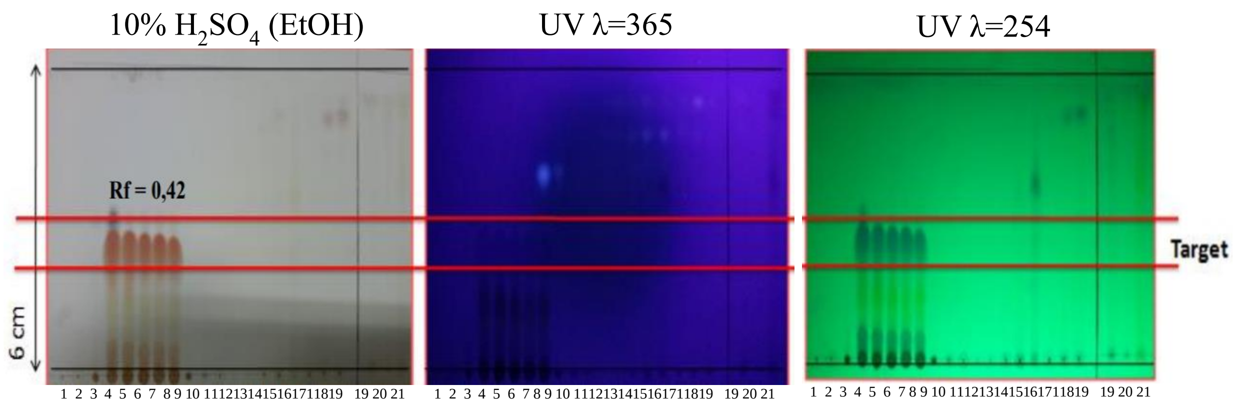

Figure S3. Chromatogram of the second separation

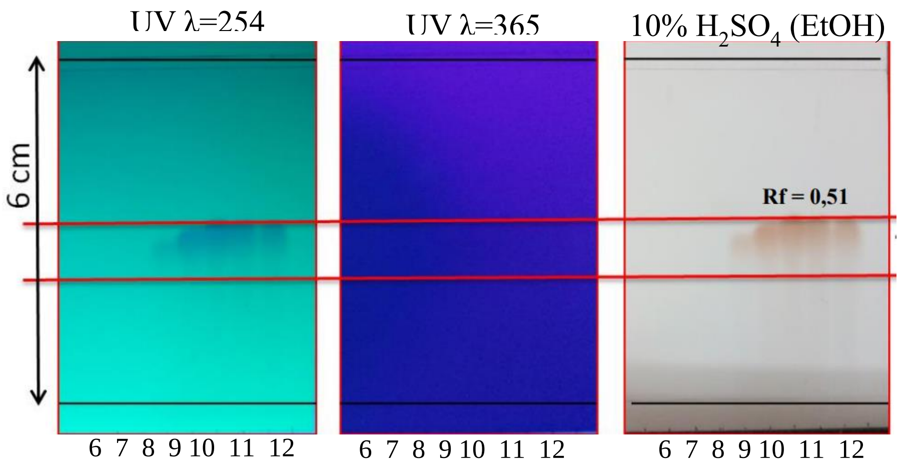

Figure S4. Chromatogram of the thrid separation
